# Dietary *Aronia melanocarpa* Pomace Supplementation Enhances the Expression of ZO-1 and Occludin and Promotes Intestinal Development in Pigs

**DOI:** 10.3389/fvets.2022.904667

**Published:** 2022-05-27

**Authors:** Zhongshuai Ren, Hengtong Fang, Jing Zhang, Rui Wang, Wenyu Xiao, Kexin Zheng, Hao Yu, Yun Zhao

**Affiliations:** State Key Laboratory for Zoonotic Diseases, Key Laboratory of Zoonosis Research, Ministry of Education, College of Animal Sciences, Jilin University, Changchun, China

**Keywords:** *Aronia melanocarpa* pomace (AMP), growth performance, gut microbiota, ZO-1, Occludin, pigs

## Abstract

A fruit juice production byproduct, *Aronia melanocarpa* pomace (AMP) is rich in natural polyphenol antioxidant components. The objectives of this study were to study the effects of dietary AMP supplementation on the feeding outcome and intestinal barrier function of pigs. In total, 27 growing pigs (Duroc × Landrace × Yorkshire, ~60 days, average weight of 27.77 ± 2.87 kg, males and females included at random) were randomly allotted to 3 treatment groups, with 3 repetitions per group and 3 pigs per repetition. At the experiment completion, 2 pigs (close to the average body weight of all experimental pigs) per replicate were slaughtered. The control group (CON group) was fed a basic diet, and the experimental groups were fed 4% (4% AMP group) and 8% (8% AMP group) AMP in the basic diet. These pigs were prefed for 3 days, and the formal experiments were performed for 7 weeks. The results showed that compared with the CON diet, the 4% AMP supplementation significantly increased the average daily gain of pigs (*P* < 0.05). Regarding intestinal development, 4% AMP significantly increased the jejunal villus height/crypt depth ratio (*P* < 0.05), and different AMP levels had no significant effect on the pig cecum morphology. Different AMP levels significantly decreased the relative abundance of Proteobacteria (*P* < 0.05). Regarding other microbial genera, 4% AMP supplementation significantly increased the levels of *Lachnospira, Solobacterium, Romboutsia* and other beneficial microorganisms (*P* < 0.05). Different AMP levels significantly decreased the relative abundances of the opportunistic pathogens *Escherichia-Shigella* and *Pseudoscardovia* (*P* < 0.05) and increased the contents of acetic acid and butyric acid in the pig cecal contents (*P* < 0.05). Compared with the CON treatment, 4% AMP supplementation significantly downregulated the jejunal gene expression of porcine proinflammatory factors (IL-1β, IL-6, IL-8 and TNF-α) and significantly upregulated the jejunal gene expression of ZO-1, Occludin and Claudin-1 (*P* < 0.05). In conclusion, 4% AMP supplementation in feed is beneficial to overall pig health and growth.

## Introduction

Pork has long been a kind of favorite meat among Chinese people. Pork accounts for approximately 60 percent of total meat consumption in China. China has a large population, and food resources such as corn, wheat and soybean meal are in short supply. There is also a lack of land and food resources needed for large-scale pig farming. Affected by the global COVID-19 pandemic, China's pig industry and the import and export of raw feed materials have been affected, and the prices of raw domestic feed materials have generally increased (1); therefore, the use of unconventional feed or agricultural byproducts to replace conventional feed in order to develop new feed resources to reduce breeding costs has become an important technological breakthrough in modern pig production.

*Aronia melanocarpa* (AM, black chokeberry) belongs to Rosaceae, a small berry native to North America (2) with edible, medicinal, landscape and other purposes (3). It was introduced into China in the 1990's; at present, it is widely planted in Northeast China, with an annual output of ~600~700 tons of fresh fruits (4). AM was approved as a new food ingredient by the National Health Commission of China in September 2018. It has been reported in many studies (5–7) that AM contains many polyphenols, such as anthocyanins, procyanidins, flavonols, and phenolic acids. The content of these compounds is generally higher than that of other fruits, so fruits and products have great potential for antioxidation and health promotion. Fresh AM peels contain many tannins, taste less astringent and are less direct to eat; most berries are used for processing to produce fruit juices and jams (8), and manufacturing these products produces a large amount of pomace. A byproduct of processing, *Aronia melanocarpa* pomace (AMP) is composed of pulp, peels, seeds and a few stems, which accounts for approximately 16% of the total amount of fresh fruit (9). The pomace contains much higher levels of polyphenol compounds than other products such as fruit juices and fruits (8, 10, 11).

Recent theoretical developments have revealed that AM and its extracts not only have obvious ameliorative effects on common diseases such as dyslipidemia, obesity (12), hypertension, bacterial infection and inflammation (13) but also inhibit the proliferation of breast, pancreatic and colorectal cancer cells (14). Currently, the large amount of AMP is directly abandoned as waste, and few studies have been conducted focusing on its utilization in pigs. Some studies have shown that dietary 2% AMP supplementation can significantly improve the average daily feed intake (ADFI) and average daily gain (ADG) and decrease the diarrhea rate of piglets (15). The gut microbiota is an important part of the intestinal microbial environment, and the biological activity of polyphenols is closely related to the gut microbiota. Gut microbiota metabolites, such as short-chain fatty acids (SCFAs), can promote intestinal development and prevent diarrhea, while disorders can induce intestinal inflammation and barrier disruption (16). In conclusion, we hypothesized that polyphenol-rich AMP could be a potential high-quality feed material and that dietary AMP supplementation might have a growth performance-promoting effect and improve intestinal health in growing-finishing pigs. Therefore, the objectives of the present study were to study the effects of dietary AMP supplementation on the feeding outcome and intestinal barrier function of pigs to provide a theoretical basis for the development and utilization of AMP.

## Materials and Methods

All experiments were approved by the Animal Protection and Utilization Committee of Jilin University, and all pigs passed inspection and quarantine and were regularly inspected and followed up by the staff.

### Animals, Diets and Experimental Design

A single factor random grouping design was used in this study. A total of 27 growing pigs (Duroc × Landrace × Yorkshire, approximately 60 days of age, average weight of 27.77 ± 2.87 kg, males and females included at random) were randomly allotted to 3 treatment groups, with 3 repetitions per group and 3 pigs per repetition. These pigs were prefed for 3 days, and the formal experiments were performed for 7 weeks. Feed consumption was recorded during the experiment. The dietary treatments consisted of the basal diet (control group, CON group) and basal diet supplemented with 4% (4% AMP group) or 8% (8% AMP group) AMP. The basic diet composition and nutritional level are shown in Table 1. Each repetition had a separate enclosure, with room temperature kept at 25~27°C and relative humidity kept at 65~75%. Determination of the nutritional composition of AMP was based on dry matter content (89.5%), crude protein (CP, 11.9%), ether extract (EE, 5.9%), ash (1.4%), crude fiber (CF, 25.6%), procyanidins (46.25 mg/g), Anthocyanins (10.56 mg/g), and gross energy (24.23 MJ/kg). The AMP used in this experiment was sourced from Jilin Province, China.

**Table 1:** Composition and nutrient levels of the basal diet [dry matter (DM) basis].

**Ingredient**	**Content, %**	Nutrient level^**^	**Content**
Corn	61.0	Digestible energy, MJ/kg	13.59
Soybean meal	26.7	Crude protein, %	17.90
Rice bran meal	8.0	Crude fiber, %	22.20
Limestone	0.9	Calcium, %	0.72
Dicalcium phosphate	1.2	Available phosphorus, %	0.70
NaCl	0.9	L-lysine, %	1.14
L-lysine	0.3	DL-methionine, %	0.27
Premix^*^	1.0		
Total	100		

* *Provided per premix kg diet: vitamin A, 5,500 IU; vitamin D_3_, 3,000 IU; vitamin E, 60 IU; vitamin K_3_, 2 mg; vitamin B_1_, 2 mg; vitamin B_2_, 4 mg; vitamin B_12_, 20 μg; vitamin B_6_, 3 mg; niacin, 25 mg; folic acid, 0.4 mg; pantothenic acid, 15 mg; biotin, 40 μg; choline chloride, 500 mg; Mn (as manganese sulfate), 15 mg; Fe (as ferrous sulfate), 80 mg; Zn (as zinc sulfate), 60 mg; Cu (as copper sulfate), 6 mg; I (as potassium iodide), 0.1 mg; and Se (as sodium selenite), 0.3 mg.*

** *Crude protein and crude fiber are the measured values, and nutrient levels were calculated. The nutritional composition of the basal diet met the National Research Council (NRC, 2012) standards*.

The pigs had free access to feed and water throughout the experiment. Each pig was weighed at the beginning and termination of the experiment to calculate the average daily weight gain (ADG). Feed intake and feed refusals per pig were recorded every day to calculate the average daily feed intake (ADFI) and the feed:gain ratio of the pigs.

### Sample Collection and Preparation

At the end of the experiment on the seventh week, all pigs were fasted for 12 h and weighed. Two pigs (close to the average body weight of all experimental pigs) per replicate were slaughtered. Blood samples were collected from the anterior vena cava of the selected pigs and centrifuged at 3,000 r/min for 15 min to collect serum. After blood collection, the same pigs were euthanized by intravenous injection of pentobarbital sodium. The intestinal tract was immediately resected and divided into the jejunum and cecum. Approximately 2 cm in the middle part of the jejunum and cecum was isolated and washed with normal saline, part of it was fixed in 4% paraformaldehyde for morphological analysis, and part of it was placed in liquid nitrogen and stored at −80°C for qPCR analysis. Finally, the contents of the cecum were quickly frozen and stored at −80°C for analysis of the gut microbiota and intestinal volatile fatty acids.

### Analysis of Serum Biochemical Parameters

The blood samples were incubated in a 37°C water bath for 10 min, centrifuged at 3,000 r/min for 15 min to collect serum, and stored at −80°C. Serum biochemical indexes included total protein (TP), albumin (ALB), globulin (GLB), urea nitrogen (urea), triglycerides (TG), total cholesterol (TC), high-density lipoprotein (HDL), low-density lipoprotein (LDL), glucose (GLU), aspartate aminotransferase (AST), alanine aminotransferase (ALT), and alkaline phosphatase (ALP) in the serum, which were measured using corresponding commercial kits (Medicalsystem Biotechnology Co., Ltd., Ningbo, China) and automatic biochemical analyzer (MS-880B, Medicalsystem Biotechnology Co., Ltd., Ningbo, China). The levels of immunoglobulin IgA, IgG and IgM were determined using commercial porcine-specific ELISA kits purchased from Nanjing Jiancheng Biological Engineering Institute (Nanjing, China).

### Serum and Jejunum Antioxidant Levels

A 10% jejunum/normal saline (0.9%) homogenate was prepared to determine the antioxidant indexes. The activity of glutathione (GSH) was detected by the colorimetric method with 5,5′-dithiobis-p-nitrobenzoic acid. The activity of total superoxide dismutase (T-SOD) was measured by the xanthine oxidase method. The malondialdehyde (MDA) level was tested as an indicator of lipid peroxidation *via* a 2-thiobarbituric acid color reaction. The catalase (CAT) activity was measured by the ammonium molybdate method. The total antioxidant capacity (T-AOC) was measured by the 2,2'-azino-bis (3-ethylbenzothiazoline-6-sulfonic acid) oxidation method. All kits were purchased from Nanjing Jiancheng Biological Engineering Institute (Nanjing, China), and the indicators were tested strictly according to the instructions of the kit.

### Intestinal Morphology

The jejunum and cecum fixed with 4% paraformaldehyde were dehydrated, embedded in paraffin and cut into 5-micron-thick slices. The sections were dewaxed and stained with hematoxylin and eosin. Five slices were obtained for each pig, and at least four images were obtained for each slice. The villus height and crypt depth were measured by ImageJ software, and the villus height/crypt depth ration (VCR) was calculated and evaluated based on intestinal morphology.

### DNA Extraction and Purification and 16S rDNA Amplification Data Analysis

After the cecum contents collected from each pig were fully dissolved and mixed separately, according to the manufacturer's instructions, DNA from different samples was extracted using an E.Z.N.A. ®Stool DNA Kit (D4015, Omega, Inc., USA). Nuclease-free water was used for the blank. The total DNA was eluted in 50 μL of elution buffer and stored at −80°C. The V3–V4 region (468 bp) of 16S rDNA was amplified using the following primers: forward primer: 5'-CCTACGGGNGGCWGCAG-3'; reverse primer: 5'-GACTACHVGGGTATCTAATCC-3'. The PCR products were confirmed by 2% agarose gel electrophoresis. Two highly variable regions of 16S rDNA, V3 and V4, were sequenced to identify the vast majority of bacteria. The PCR products were purified by 16S V3–V4 amplification, and the DNA library was constructed. An Illumina sequencer was used to sequence a qualified DNA library and obtain information for bioinformatic analysis.

After sequencing, the original data were obtained, the paired-end data were spliced with lap, the quality was controlled, the chimeras were filtered, and high-quality clean data were obtained. Instead of clustering by sequence similarity, DADA2 (divisive amplicon clustering algorithm) was used to obtain representative sequences of single-base accuracy by steps such as “de-duplication” (equivalent to 100% similarity clustering). The core of DADA2 was denoised. Then, using the concept of amplicon sequence variants (ASVs) to construct the operational taxonomic unit (OTU) table, the final feature table and feature sequence were obtained, and diversity analysis, species classification annotation and diversity analysis were carried out.

### Determination of Cecal Short Chain Fatty Acid Content

The concentrations of the main short chain fatty acids in cecal chyme were determined by gas chromatography mass spectrometry (GC–MS). First, 1 g of sample was added to 2 mL of 1:3 phosphoric acid aqueous solution, vortex homogenized for 2 min, added to 2 mL of ether for extraction for 10 min, and centrifuged at 4,000 r/min for 20 min (maintained at low temperature and placed in ice water bath). After centrifugation, the ether phase was removed, 2 mL of ether was added for extraction for 10 min again, the ether phase was removed after centrifugation, and the two extracts were combined and evaporated to a volume of 2 mL. One microliter of supernatant was injected into a gas chromatograph system for measurement (7890B-7000D, Agilent, Santa Clara, USA).

### RNA Extraction and Real-Time Quantitative Polymerase Chain Reaction

Total RNA was extracted from the jejunum. The A260/A280 nm value of RNA samples was within the acceptable range of 1.8–2.1. cDNA was synthesized using a reverse transcription kit, and quantitative PCR was performed using a SYBR Green Mix Kit (Trans, Beijing, China). Then, the prepared eight-strip tube was put into a real-time fluorescent qPCR instrument and melted at 94°C for a 30 s predenaturation, followed by thermal cycling at 94°C for 5 s, 60°C (annealing temperature) for 15 s, and 72°C and 15 s extension. The number of cycles was generally 40, and the number of cycles could be adjusted according to different primer reactions. Fluorescence quantitative results were calculated by the 2^−Δ*ΔCt*^ method to calculate the relative mRNA levels of genes. The primers used for qPCR analysis are shown in Table 2.

**Table 2:** Gene name and qPCR primer sequence^a^.

**Gene symbol**	**Primer nucleotide sequence (5' → 3')**	**Product length, bp**	**Accession No**.
β-actin	F: CTGGAACGGTGAAGGTGA R: TTTGGAAAGGCAGGGACT	218	XM_021086047.1
IL-1β	F: CCACAAATCTCTAGTGCTGGCT R: CAGGGTGGGCGTGTTATCT	199	NM_001302388.2
IL-6	F: AGGCCGTGCAGATTAGTACC R: ATTTGTGGTGGGGTTAGGGG	95	NM_001252429.1
IL-8	F: GCCTTCTTGGCAGTTTTCCTG R: TGGAAAGGTGTGGAATGCGTA	113	NM_213867.1
IL-10	F: CGGCGCTGTCATCAATTTCT R: GGCTTTGTAGACACCCCTCTC	102	NM_214041.1
TNF-α	F: TTATCGGCCCCCAGAAGGAA R: CGACGGGCTTATCTGAGGTT	102	NM_214022.1
ZO-1	F: ACCCACCAAACCCACCAA R: CCATCTCTTGCTGCCAAACTATC	123	XM_021098896.1
Occludin	F: GCTGGAGGAAGACTGGAT R: ATCCGCAGATCCCTTAAC	244	XM_005672522.3
Claudin-1	F: TACTTTCCTGCTCCTGTC R: AAGGCGTTAATGTCAATC	169	NM_001244539.1

a *IL-1β, interleukin-1β; IL-6, interleukin-6; IL-8, interleukin-8; IL-10, interleukin-10; TNF-α, tumor necrosis factor α; ZO-1, zonula occludens-1*.

### Statistical Analysis

The results are expressed as the mean ± standard deviation (SD). SPSS (version 20.0) was used to test the significance of the data by one-way analysis of variance, and *P* < 0.05 was deemed to indicate a statistically significant difference.

## Results

### Growth Performance

The growth performance results are shown in Table 3. Compared with that of the CON group, the average daily gain of the 4% AMP group was higher (*P* < 0.05). The ADFI and F/G of the 4% AMP group and the 8% AMP group were better than those of the CON group, but there was no significant difference (*P* > 0.05). No pig deaths occurred during the entire experimental period.

**Table 3:** Effect of dietary AMP supplementation on the growth performance of pigs^1^.

**Parameter^2^**	**CON Group**	**4% AMP Group**	**8% AMP Group**	***P*-value**
Initial BW, kg	28.4 ± 3.3	27.2 ± 1.25	27.73 ± 3.4	0.801
Final BW, kg	61.53 ± 3.42	64.66 ± 8.26	62.5 ± 6.85	0.226
ADG, kg	0.66 ± 0.06^a^	0.81 ± 0.05^b^	0.69 ± 0.1^ab^	0.045
ADFI, kg	1.88 ± 0.02	2.05 ± 0.04	1.93 ± 0.06	0.284
F: G	2.86 ± 0.22	2.6 ± 0.19	2.83 ± 0.41	0.396

1 *Data represent the means ± SD values of 9 pigs per treatment. ^a, b^Mean that values in the same row with different letters are significantly different at P <0.05.*

2 *CON Group, control group fed a basic diet; 4% AMP Group, basic diet + 4% Aronia melanocarpa pomace (AMP); 8% AMP Group, basic diet + 8% Aronia melanocarpa pomace (AMP); ADG, average daily gain; ADFI, average daily feed intake; F:G, feed:gain ratio*.

### Serum Biochemical Indicators

Table 4 shows that compared with the CON treatment, the addition of 4 and 8% AMP significantly decreased the TC content of pig serum and significantly increased ALP levels in pig serum (*P* < 0.05). There was no significant difference in other biochemical indexes among the three groups (*P* > 0.05). Compared with the CON treatment, the addition of 8% AMP significantly increased the IgA, IgG and IgM contents of pig serum, while the addition of 4% AMP significantly increased only the IgG and IgM contents. Compared with the 4% AMP treatment, the addition of 8% AMP had no significant effect on the contents of IgA, IgG and IgM (*P* > 0.05).

**Table 4:** Effect of dietary AMP supplementation on serum biochemical indicators of pigs^1^.

**Parameter^2^**	**CON Group**	**4% AMP Group**	**8% AMP Group**	***P-*value**
TP, g/L	66.4 ± 4.14	68.66 ± 6.28	66.31 ± 5.05	0.728
ALB, g/L	27.8 ± 3.44	28.11 ± 3.09	26.95 ± 5.42	0.899
GLB, g/L	38.6 ± 6.25	40.55 ± 5.09	39.36 ± 4.43	0.845
Urea, mmol/L	6.13 ± 0.55	6.15 ± 0.91	5.51 ± 1.27	0.498
TC, mmol/L	2.63 ± 0.13^b^	2.36 ± 0.14^a^	2.38 ± 0.16^a^	0.023
TG, mmol/L	0.56 ± 0.16	0.55 ± 0.13	0.6 ± 0.09	0.787
HDL, mmol/L	0.49 ± 0.02	0.47 ± 0.03	0.5 ± 0.06	0.679
LDL, mmol/L	1.21 ± 0.1	1.11 ± 0.15	1.18 ± 0.11	0.453
Glu, mmol/L	6.3 ± 1.29	6.18 ± 1.29	6.92 ± 1.3	0.638
AST, U/L	31.13 ± 0.62	31.8 ± 0.77	31.28 ± 1.01	0.425
ALT, U/L	52.55 ± 6.1	51.58 ± 17.14	47.45 ± 10.45	0.782
ALP, U/L	114.2 ± 6.34^a^	127.65 ± 6.13^b^	126.43 ± 8.44^b^	0.016
IgA, g/L	0.1 ± 0.01^a^	0.14 ± 0.02^ab^	0.18 ± 0.02^b^	0.039
IgG, g/L	3.1 ± 0.17^a^	4.56 ± 0.26^b^	4.45 ± 0.80^b^	0.048
IgM, g/L	2.5 ± 0.07^a^	4.17 ± 0.32^b^	4.23 ± 0.42^b^	0.002

1 *Data represent the means ± SD values of 6 samples per treatment. ^a, b^Mean that values in the same row with different letters are significantly different at P <0.05.*

2 *CON Group, control group fed a basic diet; 4% AMP group, basic diet +4% Aronia melanocarpa pomace (AMP); 8% AMP group, basic diet +8% Aronia melanocarpa pomace (AMP); TP, total protein; ALB, albumin; GLB, globulin; Urea, urea nitrogen; TC, total cholesterol; TG, triglycerides; HDL, high-density lipoprotein; LDL, low-density lipoprotein; Glu, glucose; AST, aspartate aminotransferase; ALT, alanine aminotransferase; ALP, alkaline phosphatase; IgG, immunoglobulin G; IgA, immunoglobulin A; IgM, immunoglobulin M*.

### Antioxidant Capacity

Table 5 shows that different levels of AMP had no significant effect on the T-AOC, T-SOD, CAT, GSH and MDA contents in the serum of pigs. Compared with the CON treatment, the addition of 4% AMP significantly increased the T-AOC and decreased the MDA level in the pig jejunum contents.

**Table 5:** Effect of dietary AMP supplementation on antioxidant indexes in the serum and jejunum of pigs^1^.

**Parameter^2^**	**CON Group**	**4% AMP Group**	**8% AMP Group**	***P*-value**
**Serum**				
T-AOC (mM)	0.48 ± 0.07	0.52 ± 0.06	0.41 ± 0.01	0.212
T-SOD (U/mL)	100.28 ± 3.33	100.1 ± 5.05	96.07 ± 7.63	0.714
CAT (U/mL)	7.4 ± 0.58	8.7 ± 1.5	6.16 ± 1.14	0.164
GSH (μmol/L)	5.36 ± 1.82	6.82 ± 1.37	6.34 ± 1.37	0.648
MDA (nmol/mL)	2.07 ± 1.12	1.13 ± 0.63	1.49 ± 0.52	0.536
**Jejunum**				
T-AOC (mmol/g)	0.02 ± 0.002^a^	0.07 ± 0.006^b^	0.04 ± 0.011^a^	0.001
T-SOD (U/mg prot)	173.54 ± 7.5	176.91 ± 3.63	178.5 ± 8.51	0.771
CAT (U/mg prot)	3.55 ± 1.21	3.98 ± 1.05	2.23 ± 0.33	0.235
GSH (μmol/g prot)	22.5 ± 2.72	43.49 ± 10.04	26.86 ± 9.73	0.093
MDA (nmol/mg prot)	4.65 ± 1.5^b^	1.28 ± 0.29^a^	1.01 ± 0.49^a^	0.014

1 *Data represent the means ± SD values of 6 samples per treatment. ^a, b^Mean that values in the same row with different letters are significantly different at P <0.05.*

2 *CON group, control group fed a basic diet; 4% AMP group, basic diet + 4% Aronia melanocarpa pomace (AMP); 8% AMP group, basic diet + 8% Aronia melanocarpa pomace (AMP); T-AOC, total antioxidant capacity; T-SOD, total superoxide dismutase; CAT, catalase; GSH, glutathione; MDA, malondialdehyde*.

### Intestinal Morphology

**Figure 1:**
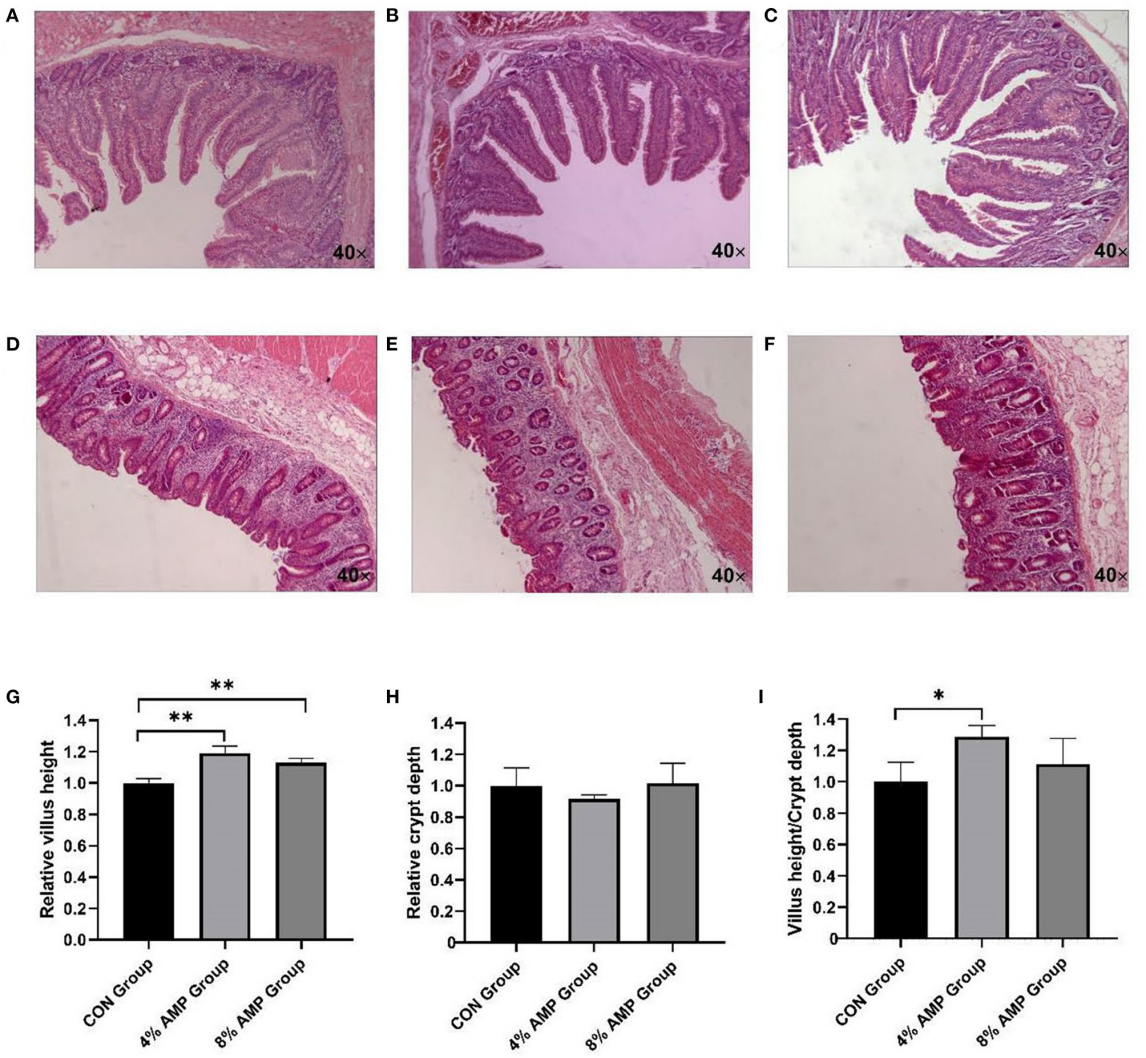
Effect of dietary AMP supplementation on the intestinal morphology of pigs. **(A)** Jejunal section of CON group pigs; **(B)** jejunal section of 4% AMP group pigs; **(C)** jejunal section of 8% AMP group pigs; **(D)** cecal section of CON group pigs; **(E)** cecal section of 4% AMP group pigs; **(F)** cecal section of 8% AMP group pigs; **(G)** jejunal relative villus length; **(H)** jejunal relative crypt depth; **(I)** villus height/crypt depth ratio (VCR). All data are expressed as the relative average ±SD, *n* = 6. *P < 0.05, **P < 0.01.

The morphological changes in the jejunum of pigs in each group were observed by staining sections (Figures 1A–C). Compared with the CON treatment, the addition of 4 and 8% AMP significantly increased the villus length of the jejunum, and the VCR was significantly increased by adding 4% AMP (Figures 1G,I). However, the addition of 4 and 8% AMP to feed had no effect on the crypt depth of the jejunum (Figure 1H). In addition, compared with the CON treatment, the addition of 4 and 8% AMP to the diet had no significant change in the morphology of the pig cecum (Figures 1D–F).

### Effect of AMP on the Intestinal Microflora

**Figure 2:**
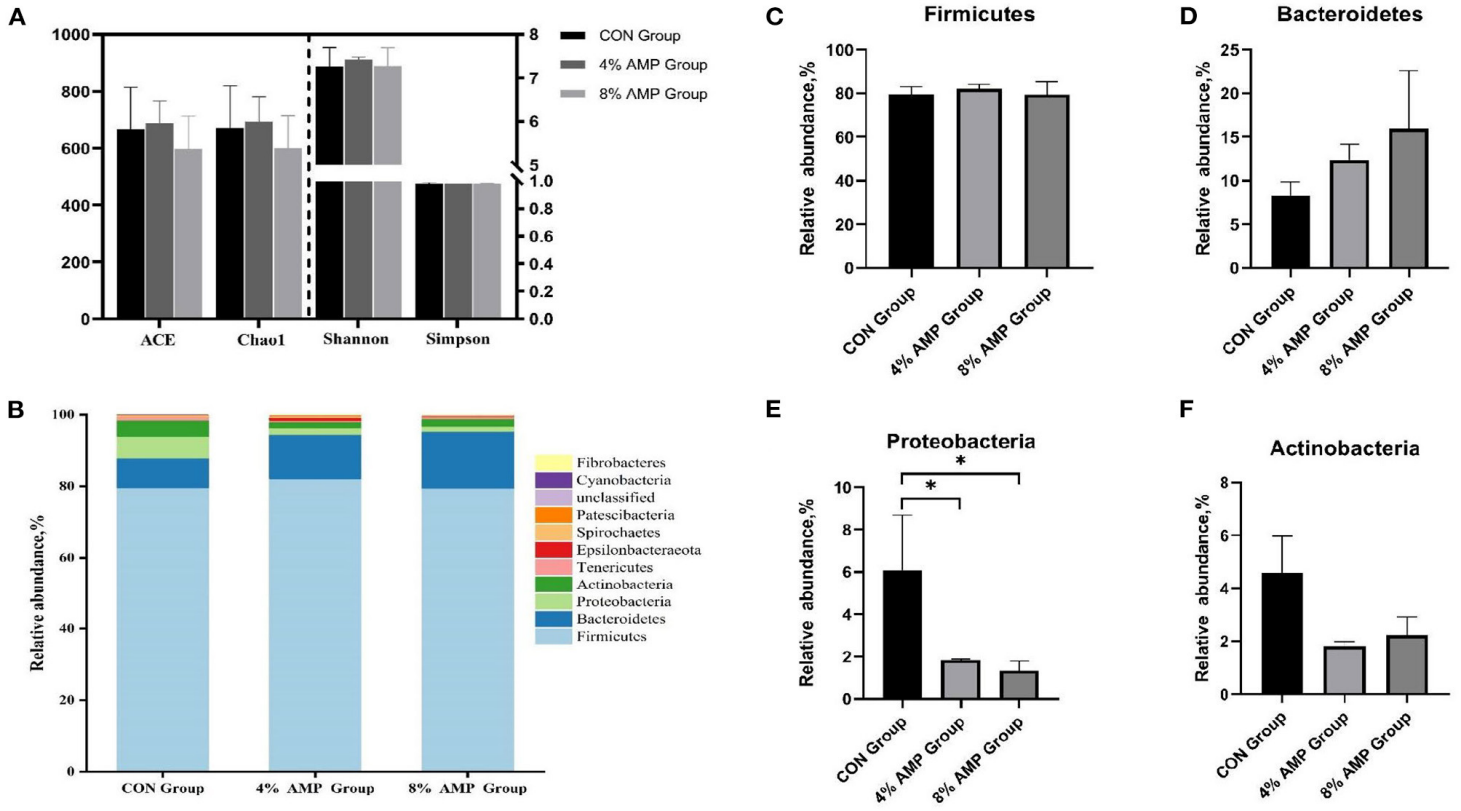
Gut microbiota regulation by AMP at the phylum level. **(A)** Species diversity and homogeneity were evaluated using the ACE, Chao1, Shannon, and Simpson indices. **(B)** Relative abundance of cecal microbial phyla. **(C)** Relative abundance of *Firmicutes*. **(D)** Relative abundance of *Bacteroidetes*. **(E)** Relative abundance of *Proteobacteria*. **(F)** Relative abundance of *Actinobacteria*. Data are expressed as the means ± SDs, *n* = 6, *P < 0.05.

As shown in Figure 2, compared with the CON treatment, dietary supplementation with different levels of AMP had no significant effect on the ACE, Chao1, Shannon, or Simpson index. At the phylum level, 11 different phyla were detected in the cecal contents of the three experimental groups. The four dominant phyla in the CON group were *Firmicutes* (79.54%), *Bacteroidetes* (8.29%), *Proteobacteria* (6.09%), and *Actinobacteria* (4.6%). In the 4% AMP group, the four dominant phyla were *Firmicutes* (82.06%), *Bacteroidetes* (12.38%), *Proteobacteria* (1.83%), and *Actinobacteria* (1.81%). In the 8% AMP group, the four dominant phyla were *Firmicutes* (79.42%), *Bacteroidetes* (15.95%), *Proteobacteria* (1.35%), and *Actinobacteria* (2.22%). The relative abundances of *Firmicutes* and *Bacteroidetes* in the CON group, 4% AMP group and 8% AMP group were 87.83%, 94.44 and 95.37%, respectively. Compared with those in the CON group, in the 4% AMP group, the relative abundance of *Firmicutes* increased by 3.17%, and that of *Bacteroidetes* increased by 49.33%; in the 8% AMP group, the relative abundance of *Firmicutes* increased by 0.15%, and that of *Bacteroidetes* increased by 92.4%, but the difference was not significant (*P* > 0.05). Compared with that of the CON group, the relative abundance of *Proteobacteria* in the gut microbiota decreased by 69.95 and 77.83% with dietary 4 and 8% AMP supplementation, respectively, and the difference was significant (*P* < 0.05). The relative abundance of *Actinobacteria* decreased by 60.65 and 51.74%, respectively, but the difference was not significant (*P* > 0.05).

**Figure 3:**
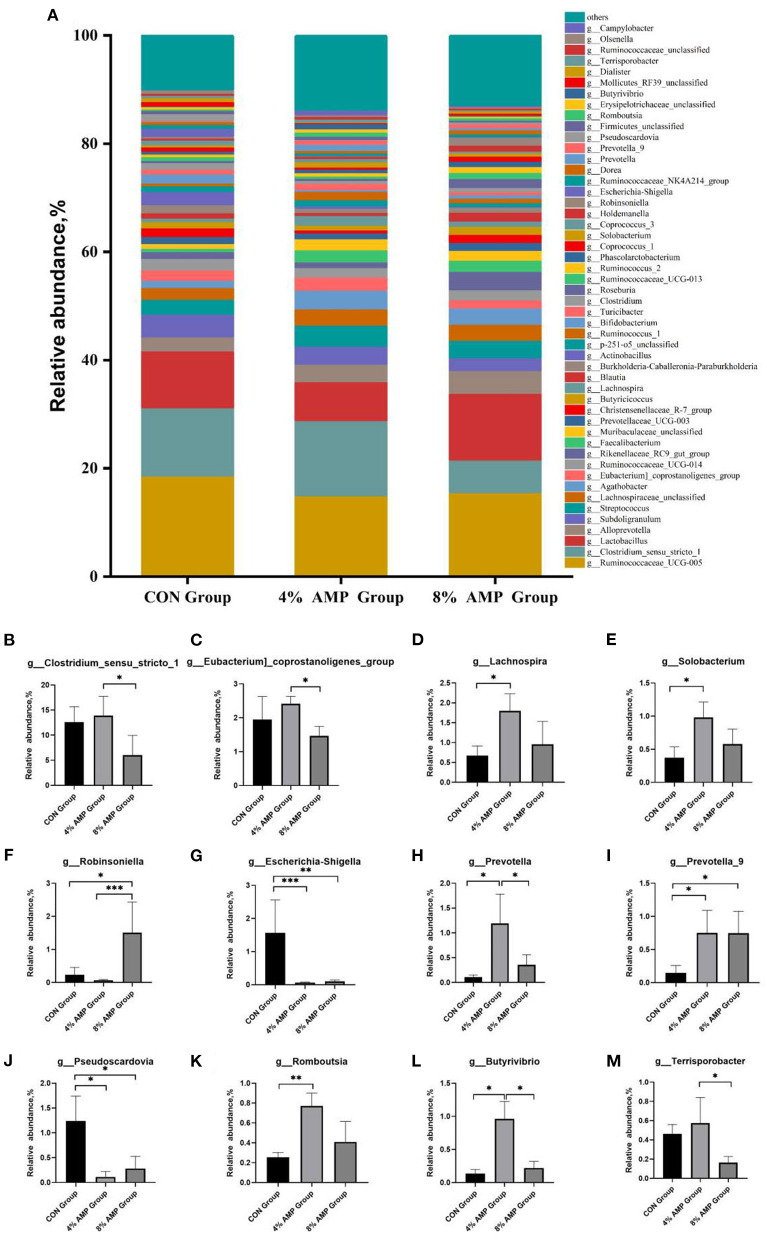
Gut microbiota regulation by AMP at the genus level. **(A)** Relative abundance of cecal microbial genera. The relative abundances of the microbial genera *g__Clostridium_sensu_stricto_1* **(B)**, *g__Eubacterium]_coprostanoligenes_group* **(C)**, *g__Lachnospira* **(D)**, *g__Solobacterium* **(E)**, *g__Robinsoniella* **(F)**, *g__Escherichia-Shigella* **(G)**, *g__Prevotella* **(H)**, *g__Prevotella_9* **(I)**, g__Pseudoscardovia **(J)**, g__Romboutsia **(K)**, g__Butyrivibrio **(L)**, and *g__Terrisporobacter* **(M)** in each group. Data are expressed as the means ± SDs, *n* = 6, *P < 0.05, **P < 0.01, ***P < 0.001.

At the genus level, a total of 245 different genera were detected. Figure 3 compares the relative abundances of the 50 most abundant genera. The five dominant genera with the highest relative abundances in the three experimental groups were *g__Ruminococcaceae_UCG-005, g__*C*lostridium_sensu_stricto_1, g__Lactobacillus, g__Alloprevotella*, and *g__Subdoligranulum*. Compared with the CON treatment, different levels of AMP treatment had no significant effect on the abundance of the above five dominant genera. Regarding other microbial genera, compared with the CON treatment, 4% AMP significantly increased the relative abundances of *g__Lachnospira, g__Solobacterium, g__Prevotella, g__Prevotella_9, g__Romboutsia*, and *g__Butyrivibrio* (*P* < 0.05) and significantly decreased the relative abundances of *g__Escherichia-Shigella* and *g__Pseudoscardovia* (*P* < 0.05). Compared with the CON treatment, The 8% AMP treatment significantly increased the relative abundances of *g__Robinsoniella* and *g__Prevotella_9* (*P* < 0.05) and significantly decreased the relative abundances of *g__Escherichia-Shigella* and *g__Pseudoscardovia (P* < 0.05). In addition, the 8% AMP treatment significantly increased the relative abundance of *g__Robinsoniella* (*P* < 0.05) and significantly decreased the relative abundance of *g__Clostridium_sensu_stricto_1, g__Eubacterium]_coprostanoligenes_group, g__Prevotella, g__Butyrivibrio*, and *g__Terrisporobacter* compared with the 4% AMP treatment group (*P* < 0.05).

### Determination of Cecal Short Chain Fatty Acid Concentrations

As shown in Table 6, dietary supplementation with different levels of AMP had no significant effect on the isobutyric acid, isovaleric acid and valeric acid contents in the cecal contents of pigs. Compared with the CON treatment, dietary 4% AMP supplementation significantly increased the acetic acid and butyric acid contents in the cecal contents of pigs, and dietary 8% AMP supplementation significantly increased the propionic acid, butyric acid and caproic acid contents in the cecal contents of pigs.

**Table 6:** Effect of dietary AMP supplementation on volatile fatty acid contents in the cecum of pigs^1^.

**Item^2^**	**CON Group**	**4% AMP Group**	**8% AMP Group**	***P*-value**
Acetic acid, μg/g	274.47 ± 3.37^a^	322.55 ± 14.51^b^	298.64 ± 11.95^ab^	0.014
Propionic acid, μg/g	338.38 ± 11.06^a^	353.75 ± 30.89^a^	427.19 ± 4.61^b^	0.008
Isobutyric acid, μg/g	20.82 ± 3.53	12.97 ± 3.89	20.63 ± 4.77	0.173
Butyric acid, μg/g	239.3 ± 22.09^a^	319.88 ± 14.75^b^	322.7 ± 20.33^b^	0.008
Isovaleric acid, μg/g	17.35 ± 1.69	10.23 ± 3.02	17.9 ± 5.1	0.133
Valeric acid, μg/g	36.28 ± 4.66	34.88 ± 3.22	34.3 ± 1.37	0.838
Hexanoic acid, μg/g	3.48 ± 0.33^a^	2.57 ± 0.36^a^	4.96 ± 0.83^b^	0.015

1 *Data represent the means ± SD values of 6 samples per treatment. ^a, b^Mean that values in the same row with different letters are significantly different at P <0.05.*

2 *CON Group, control group fed a basic diet; 4% AMP group, basic diet + 4% Aronia melanocarpa pomace (AMP); 8% AMP group, basic diet + 8% Aronia melanocarpa pomace (AMP)*.

### Gene Expression of Jejunal Inflammatory Factors

**Figure 4:**
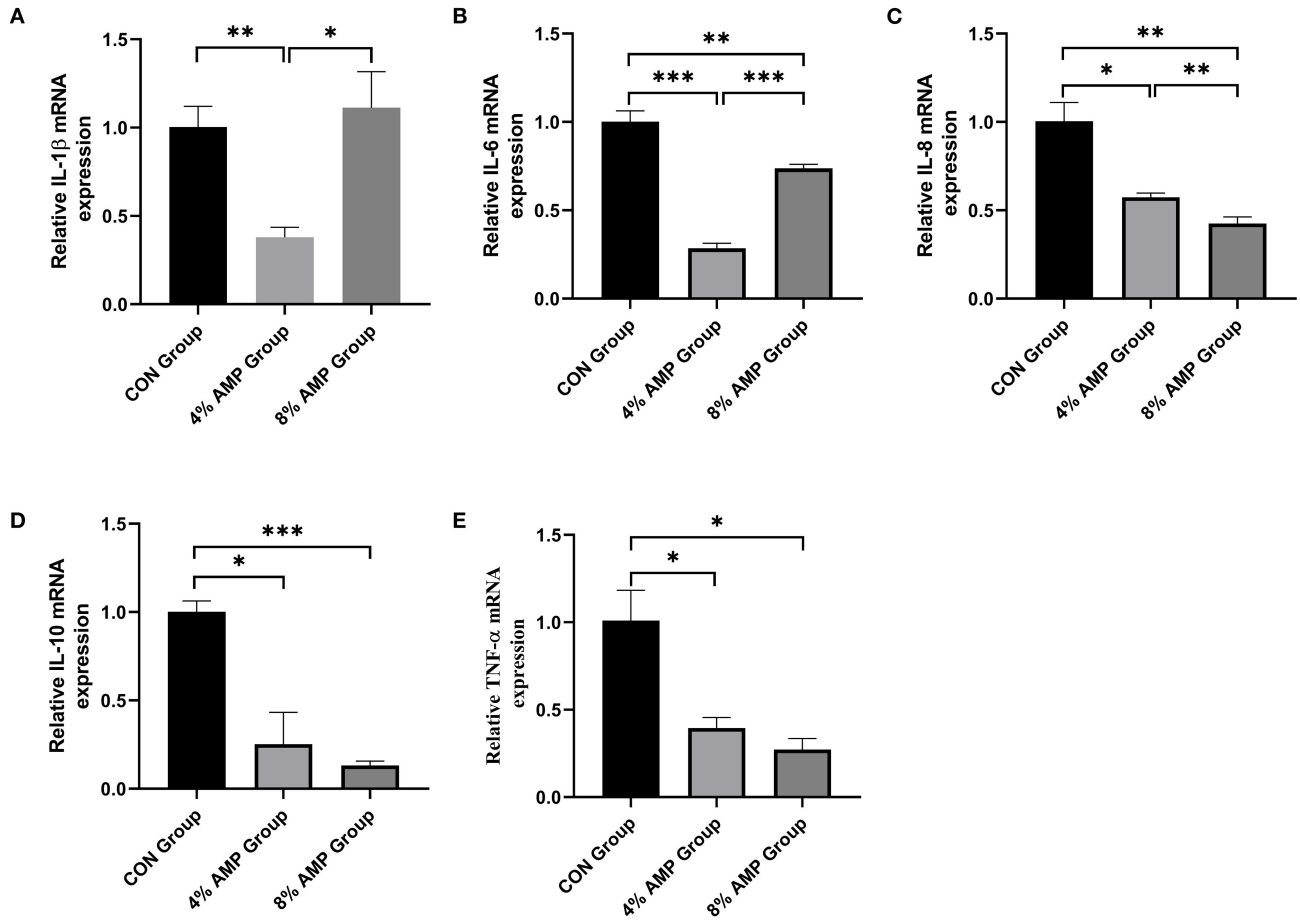
Effect of dietary AMP supplementation on the gene expression of inflammatory cytokines in the jejunum of pigs. **(A)** Interleukin (IL)-1β; **(B)** IL-6; **(C)** IL-8; **(D)** IL-10; **(E)** Tumor Necrosis Factor (TNF)-α. Data are expressed as the means ± SDs, *n* = 6, *P < 0.05, **P < 0.01, ***P < 0.001.

As shown in Figure 4, compared with the CON treatment, dietary 4% AMP significantly decreased the gene expression levels of jejunal inflammatory cytokines such as interleukin (IL)-1β, IL-6, IL-8, IL-10 and TNF-α (*P* < 0.05). Dietary 8% AMP significantly decreased the gene expression levels of the jejunal inflammatory cytokines IL-6, IL-8, IL-10 and TNF-α but had no effect on IL-1β gene expression (*P* < 0.05). In addition, compared with the 4% AMP treatment group, 8% AMP significantly increased the gene expression levels of IL-1β and IL-6 (*P* < 0.05) and significantly decreased the gene expression level of IL-8 (*P* < 0.05).

### Tight Junction Gene Expression in the Jejunum

**Figure 5:**
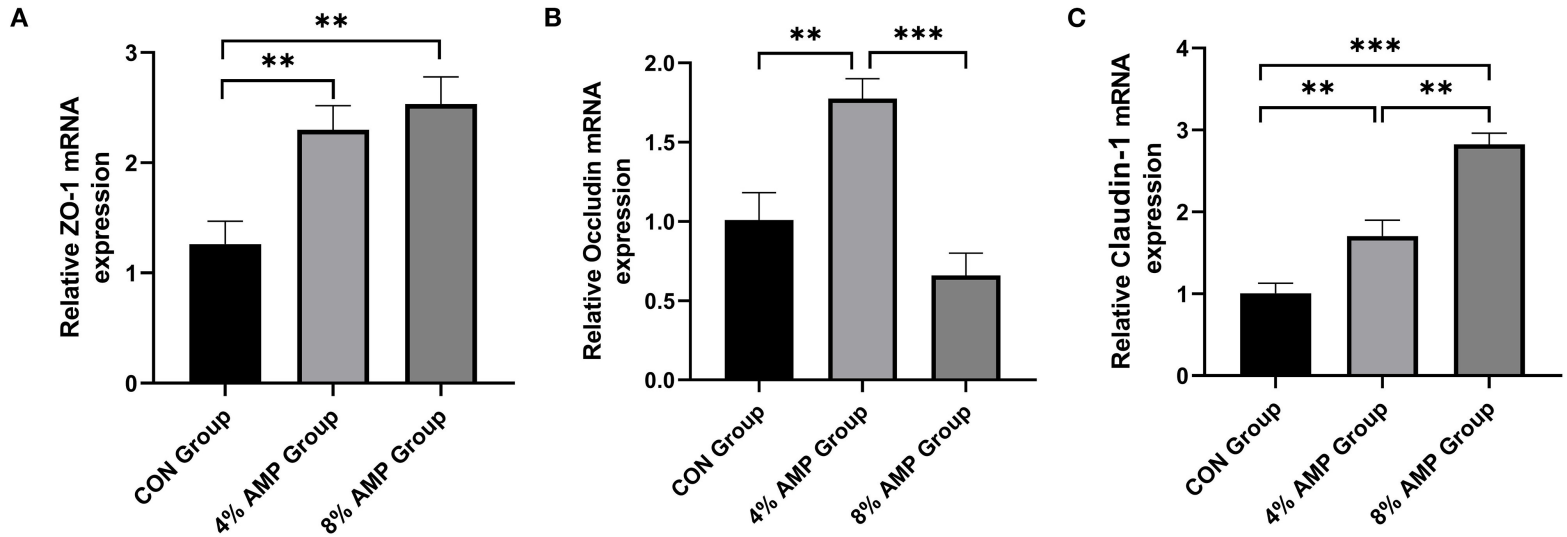
Effect of dietary AMP supplementation on the expression of genes related to intestinal barrier function in pigs. **(A)** mRNA expression of zonula occludens protein-1 (ZO-1); **(B)** Occludin; **(C)** Claudin-1. Data are expressed as the means ± SDs, *n* = 6, *P < 0.05, **P < 0.01, ***P < 0.001.

The expression levels of key genes related to intestinal barrier function are shown in Figure 5. Compared with the CON treatment, dietary 4% AMP significantly increased the expression levels of the ZO-1, Occludin and Claudin-1 genes in the pig jejunum. Dietary 8% AMP significantly increased the expression of the ZO-1 and Claudin-1 genes in the jejunum but had no effect on Occludin gene expression. In addition, compared with the 4% AMP treatment group, the 8% AMP treatment significantly increased the gene expression level of Claudin-1 (*P* < 0.05) and significantly decreased the gene expression level of Occludin (*P* < 0.05).

## Discussion

As a by-product of processing, pomace not only shows a high yield but is also rich in nutrients such as organic acids, polyphenols, vitamins, minerals, and dietary fiber. In recent years, new reports on the use of pomace to develop new feed resources to alleviate the shortage of feed resources for pig breeding have continued to emerge (17–22). Previous experiments by our research group proved that adding 2% grape pomace to the diet of weaned piglets could improve ADG, ADFI and the gain/feed ratio, but the effect was not significant (23). Sung Dae LEE et al. fed growing-finishing pigs diets supplemented with 2%-6% fermentable apple pomace for 5 weeks and found that adding 2% fermented apple pomace to the diet could significantly improve the final body weight, daily gain, feed intake and feed efficiency of pigs, while adding 4 and 6% fermented apple pomace significantly improved ADFI and pork quality (24). In this study, growing-finishing pigs (Duroc × Landrace × Yorkshire) were selected as subjects. Compared with the CON group, dietary 4% AMP supplementation significantly increased ADG and also improved ADFI and feed conversion to some extent. Compared with the CON group and the 4% AMP group, 8% AMP treatment had no significant effect on the above indexes.

The biochemical components in serum play an important role in regulating body metabolism, internal and external environmental balance and physiological function and are an important reference basis for comprehensively monitoring the overall immune level and health status of pigs as well as feeding and management (25). Serum biochemical indexes can directly reflect the metabolism and health status of the body and play an auxiliary role in the study of protein and lipid metabolism. Generally, an increase in serum TC content can induce cardio-cerebrovascular disease, while ALP content reflects liver function and bone development during the growing period (26). Some studies have shown that dietary treatment with an AM extract can significantly reduce the TC and TG contents of rat serum (27). This study revealed that 4 and 8% dietary AMP supplementation significantly decreased the TC content of pig serum and significantly increased the ALP level in pig serum but had no significant effect on the other indexes, indicating that adding different levels of AMP to feed could improve the blood health of pigs to some extent. The anthocyanins and procyanidins in AMP show strong antioxidant and anti-inflammatory activities (11). In terms of regulating the immune response of the body, Gajic et al. have shown that the oral administration of an AM extract to healthy mice upregulated the phagocytic activity of their intestinal macrophages and significantly increased the proportion of T and B lymphocytes, while *in vitro* experiments showed enhanced splenocyte viability following AM extract treatment (28). Adding 4% fermented grape dregs to the diet of weaned piglets significantly increased the IgG content in the serum of piglets (23). The results showed that different levels of AMP significantly increased the IgG and IgM contents in pig serum, and the effect of adding 8% AMP on the IgA and IgM contents was higher than that of the 4% AMP treatment.

To further measure the antioxidant effect of AMP, the effects of different levels of AMP on the antioxidant capacity of the pig serum and jejunum were also compared. The results showed that different levels of AMP had no significant effect on the T-AOC and T-SOD, CAT, GSH and MDA contents in pig serum, which may be due to the dynamic regulation of long-term polyphenol active substances in AMP. However, compared with the CON treatment, the addition of 4% AMP to the diet significantly increased the T-AOC of the pig jejunum and significantly decreased the MDA content in the pig jejunum. As an organ that was directly affected by the change in feed, the T-AOC value of the jejunum directly reflected the overall antioxidant level of the enzyme and non-enzyme systems, while the significant decrease in MDA content indicated that the antioxidant function of the jejunum was improved.

To further evaluate the health effect of dietary AMP on the growth and development of pigs, the effects of different levels of AMP on pig intestines were further studied. Villus height, crypt depth and the VCR are common indexes used to evaluate the intestinal integrity of pigs. An increase in villus height indicates an increase in nutrient absorption surface area, and an increase in crypt depth indicates rapid tissue turnover, which is usually related to a decrease in nutrient digestion and absorption capacity (29). The comparison of morphological changes in the cecum and jejunum of pigs in this experiment showed that adding 4 and 8% AMP to feed significantly increased the relative villus height in the jejunum compared with the CON group, and the ratio of jejunal relative villus height to relative crypt depth (VCR) was significantly increased in the 4% AMP group, which indicated that the effective intestinal absorption of nutrients was improved. This might be the reason why ADG increased significantly while there was no significant change in ADFI. In addition, the addition of 4 and 8% AMP to the diet had no effect on the recess depth of the jejunum, and the addition of 4 and 8% AMP to the diet had no adverse effect on the morphology of the pig cecum.

In addition to the digestion and absorption of various nutrients, the intestinal tract is also the main place for microbial colonization. There are tens of thousands of microorganisms (including fungi, viruses and bacteria) in the intestinal tract of mammals, which play an important role in maintaining the stability of the intestinal environment and the health of the host (30). Changes in feed composition may affect the composition of intestinal microorganisms. Wang et al. (31) studied the fecal microbial structure community of piglets from birth (0 days old) to market (174 days old). The results showed that *Firmicutes* and *Bacteroidetes* were the dominant microbial phyla of pigs from birth to market. The results of this study showed that at the phylum level, *Firmicutes* and *Bacteroidetes* were the two dominant microbial phyla, which was consistent with the results of Si et al. (32). At the genus level, compared with the CON and 8% AMP treatments, the 4% AMP treatment showed a more significant effect on beneficial bacteria. *g__Lachnospira, g__Prevotella, g__Prevotella_9, g__Romboutsia*, and *g__Butyrivibrio* all exhibit a positive correlation with different aspects of animal intestinal health (33–35). In addition, it is noteworthy that AM extracts inhibit biofilm formation and bacterial growth of *E. coli* and *Bacillus cereus in vitro* (36). In this study, 4% AMP and 8% AMP significantly reduced the relative abundance of *g__Escherichia-Shigella* by 95.54 and 93.63%, respectively, and reduced that of *g__Pseudoscardovia* by 91.13 and 77.42%, respectively. *Pseudoscardovia* was first found in wild pigs (37), and some studies have found that it has a significant positive correlation with liver metabolic abnormalities (38), tuberculosis (39), and other diseases and has become a marker for the early detection of colorectal cancer. The relative abundances of *g__Escherichia-Shigella* and *g__Pseudoscardovia* in the pig gut decreased significantly, which greatly reduced the risk of disease in the pig gut.

The cecum is the main site of microbial digestion, and its microbial community metabolism can produce SCFAs, provide nutrition for the animal body, participate in the energy metabolism of the host (40), promote the growth and development of the animal (41), and exert bactericidal and bacteriostatic effects. SCFAs are the final product of dietary fiber and protein fermentation by cecal microorganisms (42). The results showed that 4% AMP significantly increased the acetic acid and butyric acid contents, and 8% AMP significantly increased the propionic acid, butyric acid and caproic acid contents. Acetic acid production is associated with a variety of intestinal bacteria, most of which are anaerobic bacteria in the colon. Propionic acid is produced mainly by *Bacteroidetes* (43), and butyric acid is produced mainly by *Firmicutes*, providing a metabolic energy source for intestinal cells. SCFAs have anti-inflammatory effects, help maintain the integrity of the mucosal barrier, are associated with the regulation of the immune response, help maintain the homeostasis of intestinal microorganisms (44), and reduce the risk of colon cancer (45). Recharla's research showed that propionic acid, butyric acid and acetic acid were positively correlated with most bacteria (46). In this study, although there was no significant difference among the dominant phyla at the phylum level, at the genus level, the relative abundances of *g__Lachnospira, g__Butyrivibrio*, and *g__Romboutsia* in typical *Firmicutes* and *g__Prevotella* and *g__Prevotella_9* in *Bacteroidetes* increased significantly, greatly increasing the propionic acid and butyric acid contents, which may be related to the improvement of pig growth performance in this study.

There is a correlation between SCFA content and the relative abundance of the gut microbiota. Most gut microbiota constituents are harmless to the body, and they can directly or indirectly affect some metabolic processes of the host in a variety of ways. The SCFA pathway is one of the pathways by which intestinal flora plays a role in the host. The SCFA-GPR pathway plays an important role in regulating the immune response (47, 48). SCFAs mainly activate GPR41 (FFAR3), GPR43 (FFAR2) and GPR109. Acetic acid, butyric acid and propionic acid can bind and activate GPR41 and GPR43, but in the intestine, only butyric acid and β-hydroxybutyric acid can activate GPR109a. The addition of AMP to feed can increase the propionic acid, butyric acid and acetic acid contents and reduce the abundance of most potential pathogenic bacteria to a great extent, which may also be one of the reasons for the increase in serum immunoglobulin content. Due to the short experimental period of this study, it was impossible to more accurately explore the relationship between intestinal SCFAs and intestinal microorganisms in pigs at different growth stages, which needs to be further studied in combination with long-term *in vivo* experiments and metabonomics.

If the intestinal epithelial barrier is destroyed, the increased intestinal permeability will induce the infiltration of pathogens, toxins and antigens, which will negatively affect the absorption of nutrients (49). Typical tight junction proteins, such as ZO-1, Claudin-1 and Occludin, can enable selective permeability barriers (50). The results showed that 4% AMP significantly increased the levels of ZO-1, Occludin, and Claudin-1 gene expression in the pig jejunum, and 8% AMP significantly increased the levels of ZO-1 and Claudin-1 gene expression in the pig jejunum, which contributed greatly to the structural integrity of the jejunum. The results were consistent with the results of the histological examination of intestinal permeability. In addition, to further measure the anti-inflammatory status of the intestinal tract, the expression levels of typical proinflammatory factors (IL-1β, IL-6, IL-8 and TNF-α) were detected by *q*PCR, which are potentially related to the expression of tight junctions (51). AM extracts can reduce the IL-6, TNF-α and NO contents of LPS-stimulated macrophages (52). The results showed that 4% AMP significantly decreased the levels of IL-1β, IL-6, IL-8 and TNF-α gene expression, and the expression levels of IL-6, IL-8 and TNF-α were also significantly decreased by dietary 8% AMP, which may be partly responsible for the increased expression of ZO-1 and Claudin-1 (53). In conclusion, adding 4% AMP to basic feed was more effective in promoting the development of the pig jejunum and was more beneficial to the absorption of nutrients, helping to strengthen whole-body immunity and reducing the risk of pig intestinal diseases.

## Conclusion

In conclusion, AMP is safe to add in small amounts to pig feed as a new type of feed resource. It can improve the ADG and other growth performance indicators and the intestinal environment of pigs. Under the conditions applied in this experiment, adding 4% AMP to the feed is suggested to be most beneficial to the intestinal health and growth of pigs. The results of this study provide a theoretical basis for the application of AMP in pig feed.

## Data Availability Statement

The raw data supporting the conclusions of this article will be made available by the authors, without undue reservation.

## Ethics Statement

The animal study was reviewed and approved by Animal Protection and Utilization Committee of Jilin University.

## Author Contributions

ZR, JZ, and HF proposed the study protocol. ZR, RW, and YZ participated in the experiments. ZR, HY, and KZ contributed to the sample preparation and data analysis. ZR, JZ, HF, and WX edited and reviewed the final version of the article. All the authors provided constructive comments on the manuscript. All authors contributed to the article and approved the submitted version.

## Funding

This research was funded by the National Natural Science Foundation of China (U21A20251), the Jilin Scientific and Technological Development Program (20210202039NC), and the Changchun City Technology Extension Project of Antibiotic-Free Livestock and Poultry Production (20200000003).

## Conflict of Interest

The authors declare that the research was conducted in the absence of any commercial or financial relationships that could be construed as a potential conflict of interest.

## Publisher's Note

All claims expressed in this article are solely those of the authors and do not necessarily represent those of their affiliated organizations, or those of the publisher, the editors and the reviewers. Any product that may be evaluated in this article, or claim that may be made by its manufacturer, is not guaranteed or endorsed by the publisher.
